# College and Hospital Notes

**Published:** 1905-08

**Authors:** 


### COLLEGE AND HOSPITAL NOTES.

The Medical Department of the University of Buffalo has issued its sixtieth annual announcement, which includes the academic year of 1905-6. It is the handsomest announcement the university has yet issued and includes information of importance to every medical student. The regular term will begin Monday evening, September 25, 1905.

Hospital Appointments.—The following-named graduates in medicine of the University of Buffalo, class of 1904, have received appointments as internes:

*Buffalo General Hospital.*—Herman D. Andrews, Harry E. Braner, Stephen M. Hill, Descum C. McKenney, Victor A. Pschellas, William J. Sullivan.

*Erie County Hospital.*—William H. Prudden, Arthur C. Schaefer, Joseph A. Peaslee, Carl M. Fiero, Abraham Lande, George B. Jackson, Charles W. Bethune, Norton H. Good.

*Sisters of Charity Hospital.*—John M. Flannery, Eugene R. Linklater, George A. Becker, Edwin Carlton Foster.

*Emergency Hospital.*—George C. Fisk.

